# Perturbation Analysis of Calcium, Alkalinity and Secretion during Growth of Lily Pollen Tubes

**DOI:** 10.3390/plants6010003

**Published:** 2016-12-30

**Authors:** Lawrence J. Winship, Caleb Rounds, Peter K. Hepler

**Affiliations:** 1 School of Natural Science, Hampshire College, Amherst, MA 01002, USA; 2 Biology Department, University of Massachusetts, Amherst, MA 01003, USA; crounds@bio.umass.edu (C.R.); hepler@bio.umass.edu (P.K.H.)

**Keywords:** calcium, protons, exocytosis, tip growth, Lilium, pollen, respiration, perturbation analysis

## Abstract

Pollen tubes grow by spatially and temporally regulated expansion of new material secreted into the cell wall at the tip of the tube. A complex web of interactions among cellular components, ions and small molecule provides dynamic control of localized expansion and secretion. Cross-correlation studies on oscillating lily (*Lilium formosanum* Wallace) pollen tubes showed that an increase in intracellular calcium follows an increase in growth, whereas the increase in the alkaline band and in secretion both anticipate the increase in growth rate. Calcium, as a follower, is unlikely to be a stimulator of growth, whereas the alkaline band, as a leader, may be an activator. To gain further insight herein we reversibly inhibited growth with potassium cyanide (KCN) and followed the re-establishment of calcium, pH and secretion patterns as growth resumed. While KCN markedly slows growth and causes the associated gradients of calcium and pH to sharply decline, its removal allows growth and vital processes to fully recover. The calcium gradient reappears before growth restarts; however, it is preceded by both the alkaline band and secretion, in which the alkaline band is slightly advanced over secretion. Thus the pH gradient, rather than the tip-focused calcium gradient, may regulate pollen tube growth.

## 1. Introduction

Calcium ions are essential for pollen tube growth [1,2]. Calcium, for example, participates in cell wall structure, where it cross-links acidic pectin residues and imparts strength to the wall [3]. However, calcium is also involved with a myriad of processes within the cytoplasm where a particularly important role may be the stimulation of secretion [4,5]. It is a paradigm in both plant and animal cell biology that elevated levels of calcium stimulate secretion. With the intracellular calcium in the pollen tube being expressed as a gradient or standing wave that focuses tightly on the extreme tip of the tube where growth dependent secretion occurs [6,7], it becomes attractive to imagine that calcium stimulates secretion and growth [8].

Despite the clear spatial association between changing cytoplasmic calcium concentration and secretion, there are temporal data that fail to support this idea. Cross-correlation studies of oscillating pollen tube growth demonstrate that the increase in the intracellular calcium gradient lags the increase in growth rate by +10° to +40° [9,10]. Secretion, measured using propidium iodide (PI), a fluorescent dye that competes for calcium in binding to acidic pectins in the cell wall [11], anticipates growth by −100° [12]. When considered together it becomes apparent that calcium is out of phase with secretion by as much as 140°. These data therefore fail to support the idea that changes in calcium regulate the changes in secretion, and thus in growth. Perhaps growth dictates the increase in calcium rather than the reverse [12].

Alternatively, local pH gradients might serve as potential regulators of secretion and growth. Pollen tubes require an acidic environment (e.g., pH 4.5–6.5) for growth [13]. In addition, lily pollen tubes exhibit pH gradients in their apical domain; a slightly acidic zone occurs at the extreme tip of the tube, which overlaps with the calcium gradient, whereas back from the tip a few micrometers, there is a more prominent alkaline band that extends 5–10 µm down the shank [14,15,16]. Here, it is important to note from studies of tobacco pollen tubes, that a proton-ATPase, the enzyme responsible for the alkaline band, occurs on the apical plasma membrane, starting a few micrometers back from the tip, and thus occupies a position similar to that of the alkaline band [17,18]. Studies conducted on lily pollen tubes during normal oscillatory growth reveal that both the acidic tip and the alkaline band oscillate with the same period as the changes in growth rate [15]. While the acidic tip follows growth, much like the calcium gradient, by contrast the alkaline band reaches its high point in advance of the increase in growth rate by ~−100° [15]. The alkaline band thus possesses virtually an identical phase relationship with growth as does the process of secretion itself. 

In the work reported here we take a complementary approach to the study of linked oscillatory processes, namely perturbation analysis. Rather than quantifying oscillations in ion concentrations, wall properties and growth rate, followed by cross-correlation analysis, we used a specific cytochrome c oxidase inhibitor, potassium cyanide (KCN), to temporarily reduce the availability of ATP in the cell. Previously, we studied the role of respiration in pollen growth studies [16] confirming that several inhibitors, including Antimycin A, oligomycin and KCN rapidly shift lily pollen energy metabolism from oxidative phosphorylation to fermentation, as also reported by Dickinson [19]. Oxygen uptake ceased and fermentation, with concomitant alcohol production, continued to support pollen tube growth and oscillation for several hours. We conclude that the inhibitors were not damaging the underlying mechanisms for growth and regulation of cell wall synthesis and expansion. Their primary effect was substantial ATP limitation.

We also found that if we rapidly washed out the inhibitors the cells recovered and resumed rapid, oscillatory growth, as under control conditions. KCN was particularly effective; it washed out readily because of its solubility and volatility and entered the cells very rapidly because of its low molecular weight. Taking into account the crucial role of ATP as the energy supply for sustaining cell processes with rapid turnover rates, such as membrane ATPase to maintain proton gradients that sustain ion balance and turgor pressure [20], and acto-myosin motors for cytoplasmic streaming and vesicle transport [16], KCN offers a unique and important method to stop and start the cellular machinery of wall synthesis and expansion.

We find that even though the calcium gradient reappears before growth restarts, the return of the alkaline band is even more anticipatory. The resumption of secretion begins slightly after the increase in the alkaline band, but well before the increase in calcium. These data give further support to the idea that the alkaline band, and associated proton ATPase, play a key role in the process of secretion and in the polarized growth of the pollen tube. 

## 2. Results

### 2.1. KCN Reversibly Inhibits Pollen Tube Growth by Blocking the Electron Transport Chain

**Figure 1:**
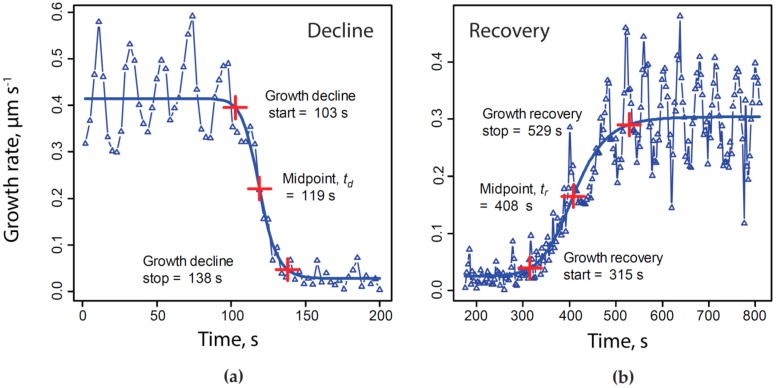
Growth response to potassium cyanide (KCN) inhibition and time base normalization—determination of a normalized time base for each experiment, based upon growth rate patterns. Four parameter logistic curves were fitted to the decline (*a*) and recovery (*b*) of growth caused by transient exposure to and removal of 200 µM KCN. The times corresponding to each frame of each time-lapse sequence were first offset to set the midpoint of growth decline (Figure 1a, *t_d_*) to zero, and then were divided by the span of growth inhibition, *t_r_* − *t_d_*. Time points corresponding to 5 and 95 percent of growth (or other measurements) range were also recorded and averaged as an estimate of the initial time, duration and completion of each phase of the response to KCN.

The general plan in this study was to first examine pollen tubes growing under control conditions, and to make sure the vital processes, notably growth, were normal. Having established a base line of activity, the lily pollen tube growth medium (LPGM) was exchanged for one that consists of LPGM plus KCN (200 µM), using a peristaltic pump. Marked growth retardation occurred after the introduction of KCN (Figure 1). It should be noted that while the growth rate declined from 0.2–0.7 µm·s^−1^ to a low level 0.02–0.05 µm·s^−1^, it did not come to a complete halt; even under maximal inhibition the growth continued to creep along at approximately one-tenth the rate of normal growth. At the point when the growth appeared to be maximally inhibited, the KCN-containing medium was removed, being replaced by normal LPGM through the peristaltic pump. Growth decline was always very rapid (33 s +/−4 s, N = 16 from beginning to end). In the early phase of recovery, the pollen tube apex nearly always first became swollen and rounded. After a brief period, the tube restarted its polarized extension, usually growing in the same direction that it had before growth was inhibited. The time needed for recovery varied (mean of 205 s, +/−52 s, N = 16) and the time from the midpoint of the decline to the midpoint of recovery varied the most (mean of 300 s, +/−91 s, N = 16). For this reason, we used the midpoint of the decline and the midpoint of the recovery as marks with which to normalize the time base for all experiments (described completely in the Methods section) so that we could objectively compare patterns of calcium, protons, secretion and energy charge during the perturbation. When growth fully recovered the growth rate routinely exhibited oscillations in rate, which were closely similar to those present before growth inhibition. 

**Figure 2:**
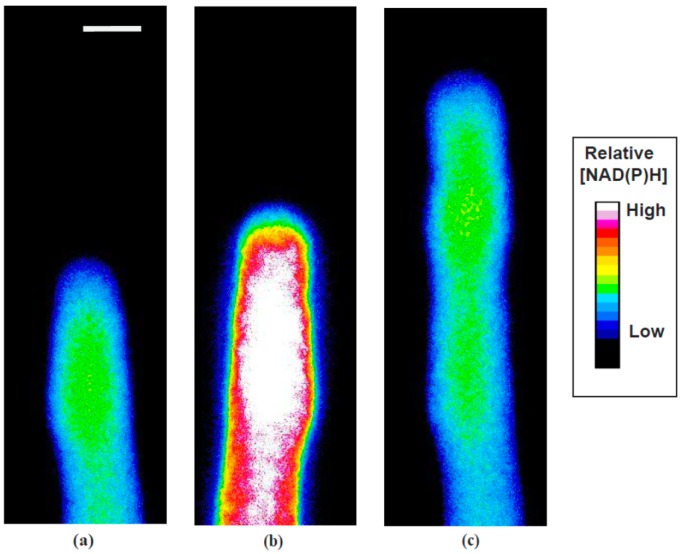
NAD(P)H; individual frames from a representative time lapse video illustrating significant spatial distributions of NAD(P)H fluorescence in a pollen tube transiently exposed to 200 µM KCN: (**a**) NAD(P)H fluorescence in an oscillatory pollen tube before KCN treatment; (**b**) maximum NAD(P)H fluorescence when growth is maximally inhibited by KCN; (**c**) return to normal NAD(P)H fluorescence after KCN is removed and oscillatory growth resumes. Bar in panel (**a**) corresponds to 10 µm.

**Figure 3:**
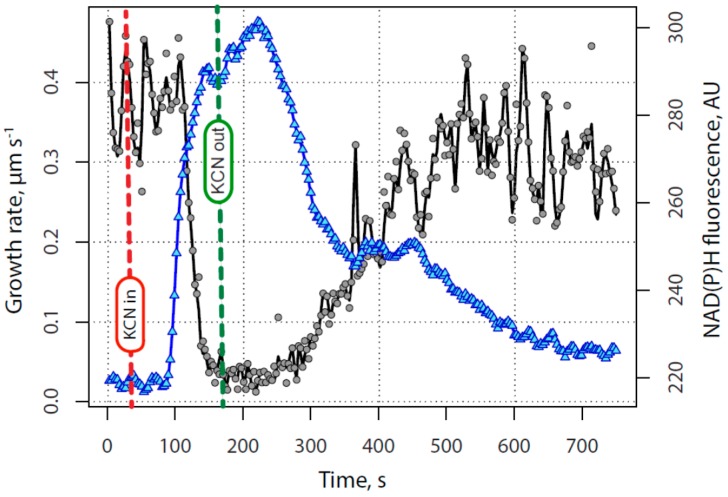
NAD(P)H time course; These representative data show the effect of transient exposure to 200 µM KCN on pollen tube growth (**black** circles) and NAD(P)H fluorescence (**blue** triangles). NAD(P)H levels rise extremely rapidly as KCN reaches the mitochondria and blocks oxidative phosphorylation. The cell is unable to regenerate electron receptors in the absence of molecular oxygen until pyruvate starts to be reduced to ethanol and the cell shifts to aerobic fermentation to supply ATP. Some of the decline in NAD(P)H levels before complete wash out of KCN may be due to aerobic fermentation. However, as soon as KCN is removed, oxidation of NAD(P)H through the electron transport chain resumes.

KCN is known to be an inhibitor of cytochrome oxidase, or site IV in the mitochondrial electron transport chain. Thus in the presence of KCN, the electron transport chain would be unable to oxidize NAD(P)H, leading to its increase, and a rapid decline in oxidative phosphorylation. Because NAD(P)H exhibits endogenous fluorescence when excited at 360 nm light and emission above 400 nm, whereas the oxidized form (NAD(P)^+^) exhibits very little, the reduced species can be examined directly in the living pollen tube [21]. Our results from repeated direct observations, a typical example of which is shown in Figure 2 and Supplemental Video 1 with analysis in Figure 3, revealed that NAD(P)H rose sharply following the introduction of KCN, likely a result of the small size and permeability of CN^−^ and its strong binding to cytochrome oxidase. 

NAD(P)H fluorescence reached a maximum after the midpoint in growth inhibition, well before growth was maximally inhibited. Growth began to decline 20 +/−6 s (N = 5) after NAD(P)H began to rise. 

Following the removal of KCN, the NAD(P)H fluorescence remained high for about 60 s, and then started to decrease after KCN was removed, with a lag similar to that of the onset of the KCN effect. The NAD(P)H signal declined slowly before growth resumed and then decreased more rapidly after the growth rate began to increase. Curiously there was typically a second, but much less dramatic, rise in the NAD(P)H fluorescence more directly correlated with the re-emergence of pollen tube growth. Taken together these data indicate that KCN is an effective and reversible inhibitor of pollen tube growth, and that its action can be explained primarily through its ability to block cytochrome oxidase and thus the mitochondrial electron transport chain, transiently limiting the supply of ATP to essential growth processes. It should be noted that KCN is also a selective inhibitor of Cu/Zn superoxide dismutase [22] and may have an effect on the ROS (reactive oxygen species) network in the cell. ROS have been shown to effect pollen germination and tube growth [23,24]. In our prior work [16,25] we explored the effects of several respiration inhibitors on pollen tube growth and NAD(P)H patterns, finding identical patterns of growth inhibition with each inhibitor. We have also found that pollen growth continues in the presence of KCN supported mainly by glycolysis, although other energy sources may also be important [26]. We conducted all of the experiments reported here with short KCN treatments to minimize the ramping up of glycolysis.

### 2.2. The Tip-Focused Calcium Gradient Reappears before Pollen Tube Growth Restarts.

**Figure 4:**
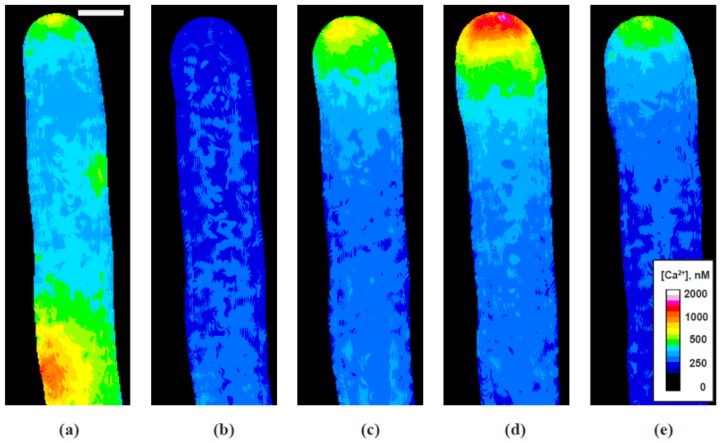
[Ca^2+^]; Individual frames from a representative time-lapse video illustrating significant spatial distributions of [Ca^2+^] in a pollen tube transiently exposed to 200 µM KCN: (**a**) calcium “spark” shortly after KCN administration and the start of growth decline; (**b**) minimum cytoplasmic [Ca^2+^] throughout the cell, corresponding with minimum growth rate; (**c**) initial establishment of the tip Ca^2+^ gradient even while growth remains minimal; (**d**) re-established active Ca^2+^ gradient during oscillatory growth, showing tip maximum [Ca^2+^]; (**e**) re-established active Ca^2+^ gradient during oscillatory growth, showing [Ca^2+^] at the tip. Bar in (**a**) corresponds to 10 µm.

**Figure 5:**
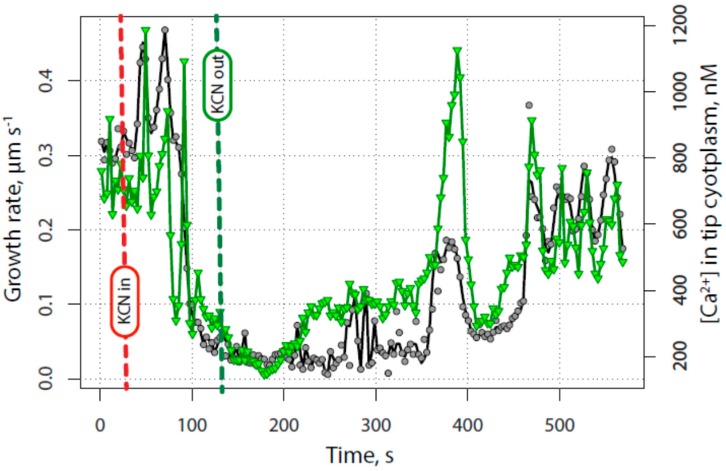
Cytoplasmic [Ca^2+^] time course—representative experiment showing the effect of transient exposure to 200 µM KCN on pollen tube growth (**black** circles) and the concentration of free calcium (as measured by the fluorescence ratio of injected fura-2 dextran) in the cytoplasm at the tube tip (**green** inverted triangles). Note that the calcium concentration follows the growth rate closely as KCN takes effect, except for a sharp spike in calcium that occurred in every experiment about 30 s before growth reached its lowest point. While growth is inhibited, calcium levels stay low (about 200 nM) until there is a sharp rise about two min after growth slows to a minimum. However, growth does not increase at this point. Eventually, growth resumes, often with an isolated burst of growth, as shown here at about 380 s, followed by a resumption of steady growth rate oscillations, at about 460 s in this experiment. Calcium peaks co-occur with growth rate peaks, typically with a short lag.

Pollen tubes that had been injected with fura-2-dextran were subjected to growth inhibition and recovery in order to determine when and where the calcium gradient reappeared following KCN removal. A representative example, shown in Figure 4 and Supplemental Video 2 with analysis in Figure 5, shows that the tip-focused gradient sharply declined together with the growth rate following the application of KCN. During the decline of tip calcium concentration and growth rate, note that there is an abrupt “spark” or “puff”, during which the calcium elevated momentarily before again resuming its decline to a low level. This event was observed in all of the cells in which calcium was measured, and in addition may be seen 50–70 µm back from the tip as shown in Figure 4a. 

Inspection of several of these events, however, has not revealed a particular spatial pattern; rather the rapid transient increases in calcium levels may occur almost anywhere in the cell. The cause of these transient calcium elevations is not known, but given the role that mitochondria play in calcium regulation [27,28], together with their sensitivity to KCN, these abrupt releases may come from mitochondria or other subcellular compartments such as the endoplasmic reticulum or vacuole. 

Additional complexity includes the observation that the calcium does not completely decline to basal levels of 150 nM; rather as both the graphs and the images show, an apical region of somewhat elevated calcium nearly always remains (Figure 5). During normal growth the maximum for the tip-focused gradient was typically 1000–2000 nM (or higher), while the basal level of calcium throughout the rest of the tube was only ~150 nM. Following treatment with KCN, together with growth inhibition, the maximum concentration of the apical calcium gradient fell to approximately 300 nM, or two-fold above the basal level of the cell.

Figure 5 further reveals that the tip-focused calcium gradient re-emerges before growth resumes. Not only does it return before growth, but it displays two stages of re-emergence. During the first phase of elevation, the calcium concentration reached an intermediate level of approximately 700–800 nM, at about 250 s in Figure 3, 100 s after the growth rate reaches its lowest value. During the second phase, it returned to the full pre-inhibition level of 1500 nM. Notice that with the second phase, strong pulses of calcium are observed, which at this time are not initially accompanied by corresponding changes in the growth rate. Only later do we see the regular oscillations in growth, which are matched by corresponding oscillations in the calcium gradient.

### 2.3. The Alkaline Band Also Appears before Growth and Anticipates Calcium

**Figure 6:**
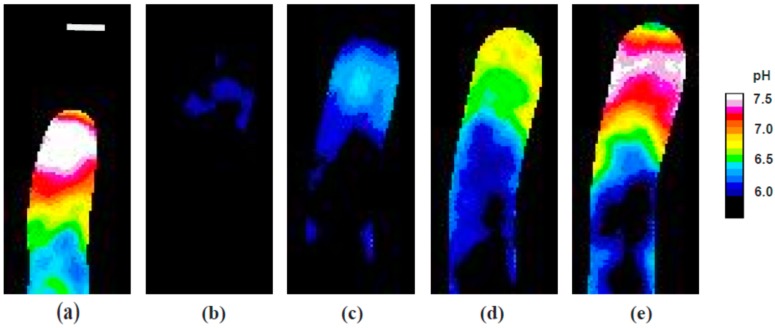
pH; Individual frames from a representative time-lapse video illustrating the dynamics of the alkaline band in response to KCN treatment, revealed by, 2′,7′-bis-(2-carboxyethyl)-5-(and-6)-carboxyfluorescein (BCECF)-dextran fluorescence ratios: (**a**) pre-KCN condition; (**b**) minimal signals indicating uniform acidification of the cell to about 6.0 as growth reaches a minimum; (**c**) proton levels begin to decline in the region that will become the alkaline band; (**d**) alkalinization at the tip; (**e**) re-establishment of the acidic tip and alkaline band, just prior to the onset of increased growth rates. Bar in (**a**) corresponds to 10 µm.

**Figure 7:**
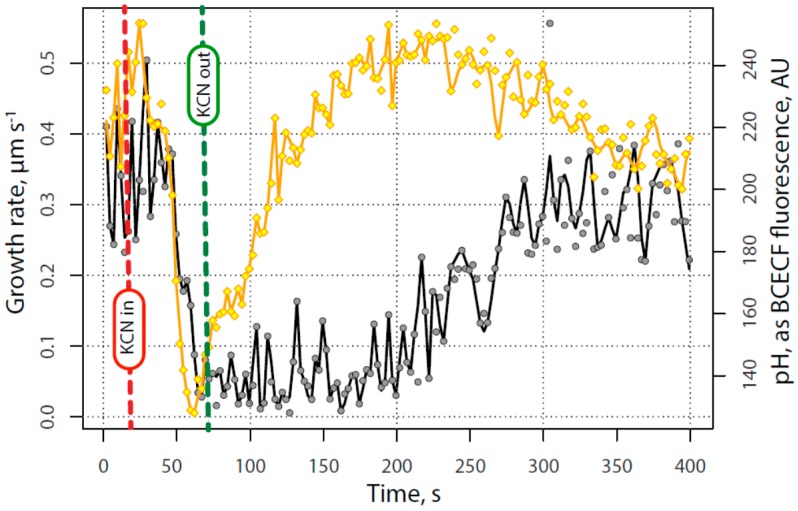
Alkaline Band time course—representative experiment showing the effect of transient exposure to 200 µM KCN on pollen tube growth (**black** circles) and the pH of the cytoplasm (**yellow** diamonds) at the locus of the alkaline band. A decrease in the pH as measured by the BCECF fluorescence ratio indicates acidification of the region that reaches a minimum just before the growth rate fully declines and before cyanide is removed. The cytoplasm begins to de-acidify immediately upon reaching a minimum and resumes its initial value close to the time that growth begins to recover. The alkaline band recovers fully about 150 s before growth is fully recovered.

When pollen tubes injected with the pH sensitive dye, 2′,7′-bis-(2-carboxyethyl)-5-(and-6)-carboxyfluorescein (BCECF)-dextran, were treated with KCN, cellular pH responded quickly, exhibiting a very rapid acidification in the region of the alkaline band (Figure 6 and Supplementary Video 3 with analysis in Figure 7).

Importantly, the alkaline band only remained at this low level briefly, and then abruptly reversed, starting to recover even before the KCN was removed. The re-emergence of the alkaline band thus precedes the increase in the growth rate by about 150 s, and precedes the first increase in calcium by about 100 s. As with calcium, a careful analysis of the images indicates detail that may be important for a more complete understanding of these processes. Whereas the pH gradient declines and reappears abruptly, the phases of re-emergence indicate first an alkalinization that occurs over a broad region of the pollen tube apex. Thereafter, the reconstruction of the normal alkaline band and acidic tip involves a narrowing and focusing of the gradient more tightly to the apex of the tube. During recovery, as noted previously, the pollen tube first swells and rounds up, and only then begins its normal polarized extension. During the rounded phase, we see that alkaline conditions are quite broadly expressed; they become more confined and restricted to the tube apex when polarized extension occurs.

### 2.4. Secretion Also Appears before Tube Growth, and before Calcium Increases

**Figure 8:**
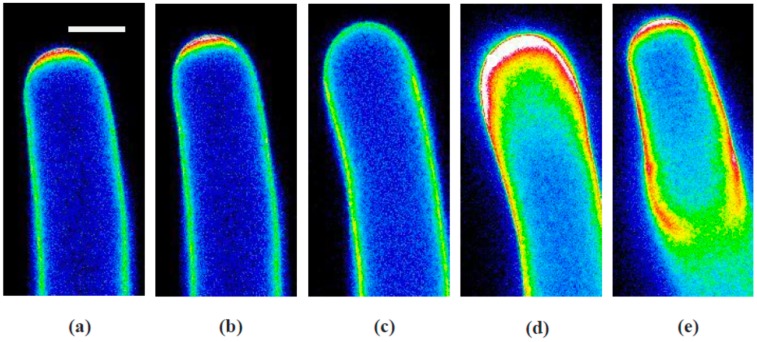
Changes in PI fluorescence at the pollen tube tip corresponding to growth rate changes caused by a transient exposure to 200 µM KCN: (**a**) oscillatory growth before KCN treatment, minimum PI signal at the tip; (**b**) oscillatory growth before KCN treatment, maximum PI signal at the tip; (**c**) minimum tip PI signal just prior to maximal growth inhibition; (**d**) large accumulation of PI-reactive material during maximal growth inhibition; (**e**) return of oscillatory growth pattern. Bar in panel (**a**) corresponds to 10 µm.

**Figure 9:**
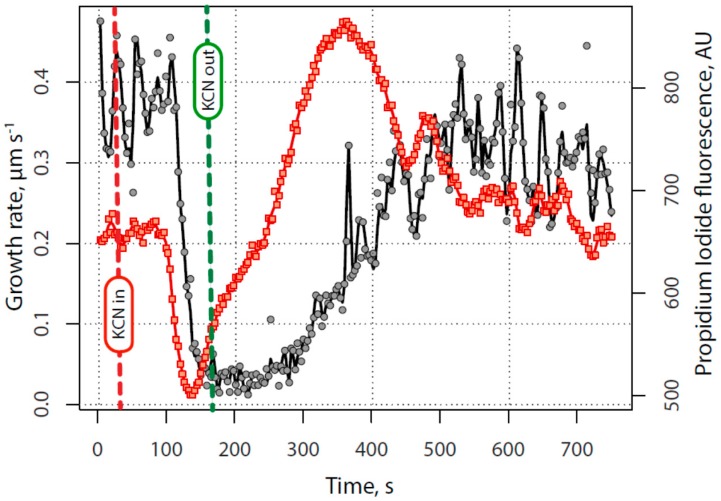
Secretion time course—representative experiment showing the effect of transient exposure to 200 µM KCN on pollen tube growth (**black** circles) and the intensity of propidium iodide (PI) fluorescence (**red** squares) at the tip of the pollen tube. Notice that the PI signal declines very rapidly and reaches a minimum even before growth reaches its minimum rate. In addition, the PI signal continues to increase, well beyond initial levels even though growth has slowed almost to zero. The PI signal reaches its maximum about half way through the recovery in growth and resumes oscillations as growth rate levels off and resumes oscillating.

When growing pollen tubes stained with PI were treated with KCN, the apical cell wall fluorescence began to decline even before the growth rate declined (Figure 8 and Supplementary Video 4 with analysis in Figure 9). 

Here, it is important to understand the meaning of this signal and its relationship to secretion. From previous work, we found that PI competes with calcium in binding to acidic pectins [11]. Because 20%–30% of the newly secreted pectins are already in their acidic form [3], they immediately become stained by PI. However, it is possible during stretching of the cell wall that the PI-stained component could be thinned out and thus give a reduced signal that is not necessarily dependent on a reduction in secretion. Another source of error could be an increase in pectin methylesterase (PME) activity, which would increase the acidic pectins under conditions in which there is no appreciable increase in secretion. For the reasons presented below, we do not think that these situations are compromising the signal that we report.

When pollen tube growth was inhibited with KCN, there was a quick and rather sharp decline in the PI signal at the tube tip (Figure 9). This signal cannot result from an excessive thinning of the wall because growth is stopping rather than increasing. It also cannot be due to increased PME activity because that would increase rather than decrease the signal. If, however, secretion is transiently reduced, perhaps due to a lack of ATP, then the normal maturation of the cell wall would continue without the addition of new PI-reactive material. The pectins present would bind calcium and the tip would take on the appearance of the wall in the tube shank, which it does.

We further argue that the marked increase in signal that then follows is due to secretion of new material rather than a modification of that which is already present, because direct microscopic inspection of the cell during this phase using differential interference contrast (DIC) reveals that the cell wall is actually thickening, indicating that new material is being delivered to the wall. From these observations, together with those from previous studies [11,29], we assert that the change in PI fluorescence is a faithful marker for the change in amount of wall material and thus indicates a change in secretion. 

Given that PI serves as a marker for secretion, the data reveal marked changes in this process during growth inhibition and recovery. Thus in a manner similar to that for the alkaline band, the PI signal first declines abruptly, reaching a low point at 10 s after the mid-point in the growth decline and in advance of the lowest growth rate (Figure 8c). However, the low level of secretion quickly reverses and starts increasing before the KCN has been removed, but with a completely different spatial pattern. While the process invariably continues upward, usually overshooting the previous values at the tip, new pectin secretion reaches well to the back of the tip in most cases, down into the shank of the tube (Figure 8d, Supplementary Video IV). The peak in tip PI signal occurs about 70 s after growth resumes. The return to normal conditions is marked by a concentration of PI fluorescence at the new point of expansion and includes the resumption of oscillations in secretion together with oscillations in the growth rate (Figure 8e).

### 2.5. Quantitative Assessment of the Relationship between Calcium, Alkalinity and Secretion with Growth

**Figure 10:**
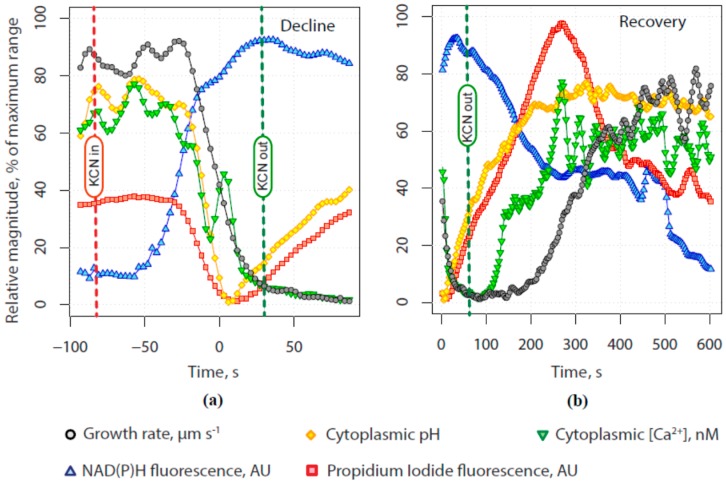
Comparison of all experiments using a common normalized time base derived from the decline (**a**) and subsequent recovery (**b**) in growth rate and relative magnitudes for each measured parameter. **Red** dashed line indicates the time that the flow of growth medium containing 200 µM KCN was started, and the **green** dashed line indicates the beginning of the flow of growth medium without KCN.

Because the methods we use to measure cellular calcium, pH and secretion are not compatible we had to acquire time series of response to transient KCN exposure from separate experiments. We found small but significant differences in the timing of growth recovery after the removal of KCN, likely due to mixing issues, so the data were difficult to average directly. Once the time base for each experiment was normalized to changes in growth rate, however, averages across five experiments of each type showed robust patterns of temporal change along a common time base as shown in Figure 10a (decline) and Figure 10b (recovery). We also fitted logistic curves to the normalized data from each experiment and so were able to determine the timing of inflection points in each signal, using 5% and 95% of maximum change as markers for the start and end of each change. These points, which we use to assess temporal differences in cellular properties are thus mathematically defined and not subject to viewer bias. All time values are reported relative to the midpoint of the decline in growth rate, set as 0 s.

During the onset of inhibition (Figure 10a), secretion (red line) and the alkaline band (orange line) both declined rapidly and at the same rate, reaching a minimum at about 10 s after the midpoint in growth decline (blue triangles). Calcium concentration (green inverted triangles) in the tube tip also declined, almost in parallel with growth rate except for a consistent burst at about the halfway point. Once KCN was washed away (20 s), both growth rate and tip calcium concentration stayed low. Even as secretion (red squares) continued to rise rapidly until 266 s, growth remained stalled until 192 s. Clearly, exocytosis was not limiting expansion at the tip. By comparison, growth did not begin to recover (192 s) until the alkaline band (yellow diamonds) was almost fully recovered. Most surprisingly, tip calcium returned to half of its initial level at 144 s, well before the resumption of growth.

## 3. Discussion

In an effort to identify factors that may be involved in the stimulation of tip growth, we have examined the return of calcium, alkalinity and secretion relative to cell elongation in pollen tubes that have been reversibly inhibited with KCN. The results show clearly that the re-emergence of the tip-focused calcium gradient precedes the increase in growth rate; however, it follows the increase in alkalinity and secretion. Nevertheless, we must acknowledge that even though the calcium is low in the apex of the inhibited pollen tubes, a small gradient remains. At about 300 nM, this may be two-fold above basal level of calcium in the shank of the tube and could possibly play a role in facilitating secretion. Regardless of these issues, the increases in calcium, first to intermediate values of 700–800 nM, and then to full recovery at 1500 nM, occur well after the increases in both alkalinity and secretion. 

Taken together, these data are consistent with those obtained from the phase relationships between calcium and secretion from oscillating pollen tubes, which show that calcium follows rather than leads the secretory process and thus is unlikely to be the stimulator of growth. We conclude, therefore, that while calcium may play an important role in pollen tube growth, it is not the agent that stimulates growth. However, calcium may still play a crucial role in sustaining polarized growth. If we assume, as has been suggested [7], that internal calcium enters from the medium, then the rapid decline in cellular concentration must be the result of a change in the balance between calcium influx and uptake by internal compartments, such as mitochondria and ER. Until growth resumes, allowing more calcium to enter, perhaps due to the opening of stretch channels [30], internal uptake keeps tip calcium low. Once growth resumes, and calcium entry increases, the tip-focused gradient is re-established. If exocytosis then follows the tip gradient, growth-induced calcium entry at the tip could be the basis for a positive feedback loop that sustains polarity as local expansion leads to focused exocytosis. 

Although calcium may be further demoted in its position as a stimulator of pollen tube growth, our data advance the case for pH, in particular the activity of the proton ATPase in generating the alkaline band. Prior evidence from oscillating pollen tubes indicates that the alkaline band and the process of secretion both increase in advance of growth by approximately −100° [12,14]. Perturbation analysis reveals that the midpoint of the return of the alkaline band occurs 180 to 200 s before the midpoint of growth recovery. Moreover, the alkaline band begins to recover almost immediately after, coincident with maximal growth inhibition, and then attains 90% of its initial pH just as growth fully recovers.

In these experiments secretion appears to be initially un-coupled from growth. Tip wall PI increases continuously and only begins to decline at about 270 s, well after growth has resumed at 190 s. It seems reasonable to interpret changes in the PI signal during growth recovery with the same model as we used during growth decline. As the cell returns to normal conditions, we are seeing a shift in the balance between exocytosis and wall expansion, such that local secretion of new wall material is exceeded by the localized expansion and thinning of the wall. 

The observations that both the alkaline band and secretion start to reverse before KCN has been removed seems contrary to our understanding of cell physiology, wherein the inhibition of the mitochondrial electron transport chain should stop all process, and likely lead to cell death. However, as we and others have shown, when the mitochondrial electron transport chain in the pollen tube is inhibited with different agents, including KCN, oligomycin, and antimycin-A, the metabolic production of energy quickly shifts from oxidative phosphorylation to glycolysis and aerobic fermentation, including the production of ethanol [16,25,31,32]. Through these metabolic processes the pollen tube is able to produce some ATP with which it keeps activities operating, e.g., cytoplasmic streaming, cell growth etc., although growth is at a lower rate. 

As a candidate for the stimulation of growth it is reasonable to ask how the pH gradient achieves its effect. From a general point of view it is important to emphasize that the proton–ATPase is the prime energetic reaction that drives ion and nutrient transport in all plant cells, including pollen tubes [33,34]. Although early reports showed considerable proton-ATPase activity associated with the pollen grain, and much less towards the tip of the tube, these results must be countered with more recent studies showing the presence of the alkaline band along the shoulder of the pollen tube apex [14]. These observations are further bolstered by those showing the presence of proton currents with an efflux on the shoulder/shank and an influx focused at the tip of the tube [14]. Finally we note the more recent studies that localize a proton-ATPase to the immediate subapical zone, which is in close proximity with the localization of the aforementioned proton currents [17,18]. Feijó et al. [14] emphasized the importance of the proton current in the pollen tube apex, in which the ATPase pumps protons out, which then flow in at the apical polar axis, creating a current loop that contributes to growth polarity. In general there is an acidification of the apical cell wall, and this may contribute to wall loosening, and stress relaxation, which promotes cell wall extension [35] (Chapter 19).

However, there may be activities in the cytoplasm that also respond to this pH gradient in ways that profoundly influence polarized cell growth. Here we refer to the possible action of the alkaline band on the closely positioned cortical actin fringe [15,36]. It has been shown in lily and tobacco pollen tubes, using improved fixation procedures, that actin is organized into a collar or fringe in the subapical domain precisely where the alkaline band occurs [37]. This fringe consists of a palisade of longitudinally oriented actin microfilaments positioned close to the cell cortex [37], likely with their barbed or fast growing ends directed toward the cell apex [38]. The actin starts one to a few micrometers back from the tip and extends down the shank another 5–10 µm. Co-localized with the actin, are two actin binding proteins, actin depolymerizing factor (ADF) and actin-interacting protein (AIP) [15]. Of particular pertinence to the present argument is the observation that ADF, perhaps especially in the presence of AIP1, responds to elevated pH (~7.5) as occurs in the alkaline band, and stimulates the local severing of the filamentous actin, and generates new plus ends, which become sites for new actin filament growth [36,39,40,41]. These conditions explain the rapid turnover of the actin fringe, which is well known for its dynamic properties [42]. 

The ionic conditions, in the presence of appropriate actin binding proteins, also account for the relative lack of F-actin in the extreme apex of the tube. Under normal pollen tube growth, when a robust tip-focused calcium gradient is present, actin microfilaments will be prevented from accumulating in the extreme apex of the tube because they will be fragmented by villin, a calcium sensitive actin binding protein found commonly in pollen tubes [43]. Not only does villin fragment F-actin, but certain isoforms will also cap the newly exposed barbed end preventing addition of new filament growth. In addition, the high calcium will cause profilin to bind and sequester G-actin, preventing it from polymerizing into F-actin [44]. 

Thus, the combined action of villin and profilin, in the presence of calcium, can account for the low level of F-actin we see at the extreme apex of the lily and tobacco pollen tube. However, only a few microns back from the tip, for example in the vicinity of the alkaline band and cortical fringe, the calcium is already much reduced from its elevated values at the extreme apex. It is our suggestion that the fringe is more strongly influenced by ADF/AIP, which participate in causing this array to exhibit its high rate of turnover. However in addition, it seems likely that there are stabilizing factors, such as the nearby plasma membrane that provides a scaffold onto which the actin forms and becomes oriented. 

It is additionally pertinent that the actin fringe is extremely sensitive, both to actin depolymerizing agents such as latrunculin B [45], and energy inhibitors such as KCN [29]. The fringe is among the first components to degrade, together with the loss of the clear zone and the inhibition of growth, but with a continuation of cytoplasmic streaming. We have further shown with KCN inhibition that secretion occurs but is no longer focused on the polar axis, and instead is spread across the apical dome. Only later during recovery from KCN does the apical actin fringe reappear concomitantly with the re-emergence of polarized tube extension [29]. A possible explanation is that the fringe prevents the docking and fusion of vesicles along the shoulders and shank of the tube, and with the plus end forward [38], the actin fringe will transport the vesicles to the cloud at the extreme apex. Given the incredible accumulation of vesicles, it may not take much to trigger or bias their fusion, possibly creating a positive feedback that facilitates further fusion and establishes the polar axis for growth.

## 4. Materials and Methods

### 4.1. Pollen Tube Growth Procedures, and Culture Chamber Construction

The procedures and solutions used in this study are very similar to those published in McKenna et al. [12], and Rounds et al. [25,29]. Briefly, plants of *Lilium formosanum* Wallace were grown from seeds in our greenhouse facility. Groups of pots, at different stages of development, are rotated through a growth/flowering phase and then into dormancy with a cold induction phase making it possible to produce flowering plants and thus fresh pollen throughout the year. When the plants flower, the anthers are harvested, from which the pollen is shaken free, and stored in 1.5 mL Eppendorf tubes in a −80 °C freezer. Our experience is that pollen viability lasts for years at −80 °C. To run an experiment, a small amount of pollen is directly placed into 1 mL of Lily Pollen tube Growth Medium (LPGM), which consists of 200 mM (7%) sucrose, 1.6 mM H_3_BO_3_, 0.1 mM CaCl_2_ and 15 mM 2-(N-morpholino)ethanesulfonic acid (MES) buffer adjusted to pH 5.7, with 10 M KOH, in a 1.5 mL Eppendorf tube. This is then clamped onto a rotator for approximately an hour by which time the pollen grains have germinated, and extended their tubes. 

The young pollen tubes are then plated onto slide chambers, designed for use with an inverted light/fluorescence microscope (Nikon Eclipse TE 300, Melville, NY, USA). The chamber was constructed by starting with a 75 × 50 mm microscope slide through which a 20 mm hole has been drilled. A 25 mm square, number 1 cover glass was then glued across the hole using nail polish (Sally Hansen, Hard as Nails, New York, NY, USA). To prepare for the culture and inspection of pollen tubes, this chamber was cleaned with soap and water. To adhere the pollen grains and tubes to the cover glass surface, we first applied a drop of warm (~ 40 °C) low gelling Agarose, type VII (Sigma, St. Louis, MO, USA). Then, an equivalent drop of pollen culture medium with tubes was gently mixed with the still warm Agarose, quickly spread over the cover glass surface, and finally cooled to gel the Agarose. Chambers thus prepared can be used for hours of observation (for details, see [12]).

### 4.2. Cell Observation

Pollen tubes were examined using a Nikon Eclipse TE300 inverted light microscope, equipped with a 40X (1.3 n.a., oil immersion) objective lens. Image capture was made using a charge-capture device (CCD) camera (Quantix Cool Snap HQ, Roper Scientific, Vianen, The Netherlands). Fluorescence excitation light was provided by a 175 watt ozone-free xenon lamp in a DG-4 housing and wavelength switching assembly (Sutter Instruments, Novato, CA, USA). To examine intracellular calcium, the pollen tubes were injected with fura-2-dextran (10 kD), with excitation at 340 nm (high calcium) and 380 nm (low calcium), and emission above 520 nm (for details of microinjection procedures, see Vos et al. [46]). For pH, the tubes were injected with BCECF-dextran (70 kD), with excitation at 440 nm (reference λ) and 490 nm (pH sensitive), and emission above 520 nm. Because pH gradients can be readily dissipated if the dye concentration becomes elevated, we strived, using the methods devised by Feijó et al. [14], to keep the BCECF at or below 1 µM. However, because of the low dye concentration, it was necessary to bin the image 3 × 3 in order to have sufficient signal. 

For secretion, the cells were stained with propidium iodide (PI) (20 µM), with excitation at 535 nm and emission at 615 nm. The energy poise of the tubes was monitored by measuring the endogenous fluorescence from NAD(P)H, with excitation at 360 nm and emission above 400 nm. All of the associated equipment including filter wheels, camera shutters, exposure times, etc. was controlled by MetaMorph/MetaFluor software (ver.6.0, Molecular Devices, Sunnyvale, CA, USA). For DIC imaging, a polarizing filter was mounted in a filter wheel (Sutter Instruments λ10-2, Novato, CA, USA) and positioned before the CCD camera. 

### 4.3. Growth Inhibition and Recovery

To perform the growth inhibition and recovery, we first monitored an appropriately labeled cell under control conditions to insure that it exhibited normal growth behavior, and then treated it with LPGM plus freshly prepared KCN (200 µM). This was achieved using a two-tube peristaltic pump; one tube was used for adding medium with or without KCN, while the second tube was used to remove medium from the slide chamber. The flow rate was set at 0.5 mL·min^−1^; because the volume of the culture chamber is only 0.3 mL, the full exchange of fluids occurs quite quickly. Pollen tubes being treated with KCN were closely monitored, so that when the growth rate reached its low level, the inhibitor was promptly washed out using the peristaltic pump to introduce fresh LPGM.

### 4.4. Normalizing Time Series to a Common Time Base

Because we wished to compare time courses of changes in calcium, alkalinity and energy charge from different experiments with a resolution of seconds we had to develop a method of normalizing each experiment to a common time base. We found that although the exact timing of growth decline following cyanide addition varied from experiment to experiment, the pattern of steep decline followed by very low to no growth and a slow recovery was observed with every cell. Consequently, we have chosen to use changes in the rate of tube extension, measured with great care in each experiment, in order to establish two benchmarks that were then used to normalize the dynamics of other cell properties. 

As shown in Figure 1, we used non-linear regression to fit the initial decline in growth to a four parameter logistic curve (Equation 1), and then determined the midpoint of that decline:

(1) $$G=\left(\frac{A-D}{1+{\left(\frac{t}{C}\right)}^{B}}\right)+D$$

where *t* = elapsed time, s;
*G* = growth rate, µm·s^−1^;
*A* = maximum asymptote of growth rate, µm·s^−1^;
*B* = Hill slope;
*C* = inflection point;
*D* = minimum asymptote of growth rate, µm·s^−1^.

We used the time of the midpoint of the growth rate decline as the initial benchmark, *t_d_*. Then, we used the same regression method to determine the midpoint of growth recovery, *t_r_*. In each experiment we then subtracted t_d_ from the values for each time point, setting the midpoint of growth decline to zero. Offset time values were divided by (*t_r_* − *t_d_*) thus expressing changes in growth, pH, calcium concentration and NADH as a function of the fraction of the total elapsed time for growth decline and recovery. So that we could determine actual time between events, the fractional time values for elapsed time were multiplied by the mean of all the total elapsed times for changes in growth from all experiments:

(2) $$t_{n}=\frac{\bar{t}\left(t-\;t_{d}\right)}{\left(t_{r}-\;t_{d}\right)}$$

$t_{n}$ = time normalized to growth decline and recovery, s;
t = actual elapsed time during experiment, s;
$t_{d}$ = midpoint of KCN-induced growth rate decline, s;
$t_{r}$ = midpoint of growth rate recovery following KCN removal, s;
$\bar{t}$ = mean of $\left(t_{r}-\;t_{d}\right)$ across all experiments included in our analysis, e.g., average duration of growth decline due to exposure to 200 µM KCN, s.

## 5. Conclusions 

We transiently exposed rapidly growing lily pollen tubes to 200 µM KCN thus markedly slowing the growth and causing the associated gradients of calcium and pH to sharply decline. We quickly washed out the KCN so that we could observe the timing of the re-establishment of ion gradients, and of exocytosis. Oscillatory growth and other vital processes fully recovered over several minutes. We found that the calcium gradient reappears before growth restarts; however, it is preceded by both the alkaline band and by secretion, in which the alkaline band is slightly advanced over secretion. Thus the pH gradient, rather than the tip-focused calcium gradient, may regulate pollen tube growth. 

## Supplementary Materials

The following are available online at www.mdpi.com/2223-7747/6/1/3/s1, Video S1: NAD(P)H, Video S2: Cytoplasmic calcium, Video S3: Cytoplasmic pH, and Video S4: Propidium iodide.
