# Supplementary material for: Perturbation Analysis of Calcium, Alkalinity and Secretion during Growth of Lily Pollen Tubes
Source: Plants (Basel). 2016 Dec 30;6(1):3. doi: 10.3390/plants6010003 (PMC5371762; doi:10.3390/plants6010003)
Supplement: Supplementary file 1 [file plants-06-00003-s001.zip › plants-161701-supplymentary.pdf]

Supplementary Materials: Perturbation Analysis of Calcium, Alkalinity and Secretion during Growth of Lily Pollen Tubes

Lawrence J. Winship, Caleb Rounds and Peter K. Hepler

Supplemental Videos:

Video S1. Changes in the spatial distribution of combined reduced nicotinamide adenine dinucleotide and reduced nicotine adenine dinucleotide phosphate (NAD(P)H) fluorescence in actively growing, oscillating lily (*Lilium longiflorum*) pollen tubes, when transiently exposed to 200 μM potassium cyanide (KCN). Frame grabs (Figure 2) and time series analysis (Figure 3) in text. These are not ratio images, so an undetermined part of the pattern across the cell is due to its cylindrical shape. White scale bar corresponds to 10 μm. Calibration of colors as in Figure 2. KCN exposure marked by "KCN" label.

Video S2. [Ca^{2+}] Changes in the spatial distribution of calcium concentration in the cytoplasm of actively growing, oscillating lily (*Lilium longiflorum*) pollen tubes, when transiently exposed to 200 μM KCN, as determined by the fluorescence ratio of injected fura-2 dextran. Frame grabs (Figure 4) and time series analysis (Figure 5) in text. White scale bar corresponds to 10 μm. Calibration of colors as in figure 4. KCN exposure marked by "KCN" label.

Video S3. pH Changes in the spatial distribution of protons in actively growing, oscillating lily (*Lilium longiflorum*) pollen tubes, when transiently exposed to 200 uM KCN, as determined by ratio fluorescence imaging of 2',7'-bis-(2-carboxyethyl)-5-(and-6)-carboxyfluorescein (BCECF)-dextran. Frame grabs (Figure 6) and time series analysis (Figure 7) in text. White scale bar corresponds to 10 μm. Calibration of colors as in Figure 6. KCN exposure marked by "KCN" label.

Video S4. PI Changes in propidium iodide (PI) fluorescence in the cell wall of actively growing, oscillating lily (*Lilium longiflorum*) pollen tubes, when transiently exposed to 200 μM KCN. Frame grabs (Figure 8) and time series analysis (Figure 9) in text. White scale bar corresponds to 10 μm. KCN exposure marked by "KCN" label.
